# Application of Nano-Drug Delivery System Based on Cascade Technology in Cancer Treatment

**DOI:** 10.3390/ijms22115698

**Published:** 2021-05-27

**Authors:** Ying Sun, Xiaoli Ma, Hao Hu

**Affiliations:** 1 Institute of Biomedical Materials and Engineering, College of Materials Science and Engineering, Qingdao University, Qingdao 266071, China; sunying150996@163.com; 2 Qingdao Institute of Measurement Technology, Qingdao 266000, China; maxiaoli1989@yeah.net

**Keywords:** combination therapy, cascade technology, multidrug resistance, tumor microenvironment response

## Abstract

In the current cancer treatment, various combination therapies have been widely used, such as photodynamic therapy (PDT) combined with chemokinetic therapy (CDT). However, due to the complexity of the tumor microenvironment (TME) and the limitations of treatment, the efficacy of current treatment options for some cancers is unsatisfactory. Nowadays, cascade technology has been used in cancer treatment and achieved good therapeutic effect. Cascade technology based on nanotechnology can trigger cascade reactions under specific tumor conditions to achieve precise positioning and controlled release, or amplify the efficacy of each drug to improve anticancer efficacy and reduce side effects. Compared with the traditional treatment, the application of cascade technology has achieved the controllability, specificity, and effectiveness of cancer treatment. This paper reviews the application of cascade technology in drug delivery, targeting, and release via nano-drug delivery systems in recent years, and introduces their application in reactive oxygen species (ROS)-induced cancer treatment. Finally, we briefly describe the current challenges and prospects of cascade technology in cancer treatment in the future.

## 1. Introduction

In recent years, nanotechnology has been widely used in the field of pharmaceutics as a method to overcome multidrug resistance (MDR), improve biodistribution and limit toxicity [1,2,3,4]. The prognosis based on the conventional combination method is unsatisfactory, mainly due to the poor specificity of chemotherapeutic drugs and the biological complexity of the tumor. The complexity, diversity, and heterogeneity of tumors have severely destroyed the therapeutic potential of treatment [5]. The complex drug resistance mechanisms of tumor cells include changes in apoptosis pathways, DNA repair and damage responses, changes in metabolic conversion of drug targets, and self-factors such as higher interstitial fluid pressure (IFP) and dense extracellular matrix (ECM) [6]. These are obstacles to the treatment. Fortunately, nanocarriers have outstanding performance in improving tumor targeting, controlled release, and pharmacokinetics [7]. The use of nanocarriers to deliver small-molecular chemotherapeutic drugs is a commonly used method in the clinical treatment of cancer. At present, the commonly used nanocarriers in clinical research mainly include liposomes, micelles, hydrogels and peptides, etc. These nanocarriers provide chemotherapeutic drugs for targeted treatment of cancer and improve the pharmacokinetics and biodistribution. In addition, new treatment methods based on functional materials with unique physicochemical properties have emerged in large numbers to achieve more accurate, efficient, and non-invasive cancer treatment. For example, photosensitizers (PSs) have excellent light absorption and conversion abilities, which can convert exogenous light energy into hyperthermia for photothermal therapy (PTT) or catalyze the production of reactive oxygen species (ROS) for photodynamic therapy (PDT) [8,9,10]. More interestingly, the tumor microenvironment (TME) responsive nano-drug delivery systems (DDS) based on the complex characteristics of TME, such as acidic pH, reduction conditions, hypoxia, hydrogen peroxide (H_2_O_2_) overexpression, have been used in tumor specific therapy. For instance, smart upconversion nanoparticles (UCNPs) can make use of various biophysical and chemical characteristics of TME to achieve precise tumor targeting [11,12].

In recent years, drug combination therapy or drug cocktail therapy has been extensively exploited and is increasingly becoming the standard practice for combatting cancer [13]. Combination therapy can be sorted into different categories, such as the combination of two chemotherapeutic drugs, the combination of chemotherapeutic drugs and nucleic acid drugs, and the combination of near-infrared light (NIR)-responsive drugs and photothermal therapy. These combined methods can effectively overcome the drug resistance of tumors, and they achieve better anticancer effects than single therapy. However, in combination therapy, different modes of administration will affect the effect of combination therapy. Although the combination of two or more drugs can usually produce synergistic therapeutic effect, the simultaneous administration of drugs may bring new problems. The possibility of drug interactions makes it difficult to deliver two or more drugs at the same time. Multiple drugs administered at the same time may aggravate adverse reactions and limit the maximum therapeutic effect of one or more drugs [14,15]. In addition, the poor correlation and synergy between different treatment methods may make the effect of combination therapy worse in magnifying the treatment results.

In this regard, cascade design is introduced into cancer treatment through nanoplatform, which can reduce adverse reactions and show great potential in cancer treatment. Cascade technology refers to a series of events that occur in sequence, or to a case in which the previous event has a promoting effect or amplification effect on the subsequent events. At present, cascade technology has been widely used in biomedicine and has achieved good performance in cancer treatment. For example, the PS, phophorbid A (PhA), was conjugated to water-soluble glycol chitosan (GC) via a ROS-sensitive thioketone (TK) linker. When nanoparticles (NPs) reach the ROS-rich hypoxia core of tumor tissue, they release PS in the photoactive form, through effective and ROS-sensitive TK bond cleavage, resulting in strong phototoxicity. After irradiation with NIR, the local ROS level increased, which promoted PS release subsequently [16]. These cascade reactions lead to a significant reduction in tumor volume. Additionally, cascade delivery refers to the nanosystem transporting across multiple biobarriers via the transition of its characteristics. Under specific TME conditions and/or external stimulation, cascade responsive nanocarriers can realize multi-step localization or multi-stage trigger release of the load of therapeutic drugs in tumor cells (even in specific organelles), minimize side effects and enhance their bioavailability. Up to now, substantial progress has been made in the study of nanoformulations for cascade anticancer therapy. Compared with the control nano-preparation, integrating targeted nanomedicines with cascade therapy further enhances tumor accumulation/penetration, facilitates cell internalization and lysosomal escape, controls intracellular drug release, protects instable therapeutics, induces synergistic effect, ameliorates therapeutic efficacy, diminishes the drug resistance and mitigates the side effects.

In the following, we first described the mechanism of drug resistance. Then, we summarize the application of cascade technology in cancer treatment which can be divided into cascade delivery and cascade reaction from the different forms and uses of cascade reaction. Finally, we discuss the challenges and application prospects of cascade technology in cancer treatment, in order to stimulate more innovative thinking and promote the rapid development of anticancer treatment.

## 2. Multidrug Resistance of the Tumor Microenvironment

MDR is the biggest obstacle facing cancer treatment, which greatly impacts and limits the therapeutic efficacies and outcomes. Tumor cell interactions with TME are crucial in epithelial-mesenchymal transition (EMT) and MDR [17]. Intracellular resistance includes the overexpression of drug efflux pumps, the induction of cell survival pathways, and the inability to induce apoptosis [18]. ATP-binding cassette (ABC) transporter proteins are mainly responsible for the drug efflux. Multidrug-resistance protein 1 (MDR1)/permeability-glycoprotein (P-pg)/ABCB1, MDR-associated protein 1 (MRP1) and breast cancer resistance protein (BCRP)/ABCG2 are the most studied ABC transporters [19].

The constant interactions between tumor cells and the TME play decisive roles in tumor initiation, progression, metastasis, and response to therapies [20]. The composition of the TME varies between tumor types, but hallmark features include immune cells, stromal cells, blood vessels, and the extracellular matrix [21]. TME is not just a silent bystander, but rather an active promoter of cancer progression [22]. MDR not only presents at the cellular level, but also at the level of TME [18]. The environment-mediated drug resistance is a result of continuous crosstalk between the tumor cells and their surrounding stroma [23]. TME plays an important role in effective drug delivery, so its drug resistance cannot be ignored when aiming to develop better treatments for resistant cancers. Abnormal vascular system and cell adhesion are the culprits of microenvironmental MDR mechanism. Among them, the abnormal vascular system causes fewer nutrients and less oxygen to be transported to cancer cells. Hypoxia can lead to the upregulation and activation of the heterodimeric transcription factor hypoxia-inducible factor-1 (HIF-1), which triggers multiple signaling pathways and leads to the occurrence of the MDR phenotype [18]. Cell adhesion-mediated drug resistance (CAM-DR) is mainly due to the attachment of tumor cells to the stroma, which may trigger several signal transduction pathways, resulting in reduced sensitivity to anticancer therapies [23]. Clinical studies have shown that the three families of cell–cell interaction molecules are selectins, Siglecs, and integrins, and they have been extensively studied in the research of inhibiting cell adhesion as a therapeutic target [24]. Selectins are C-type lectins that bind to properly modified glycan ligands, such as CD44, Eselectin ligand-1, CD43, CD34, or addresses with a variable specificity. For example, the study by Hao et al. showed that both CD147 and CD44 are involved in cancer drug resistance [25]. CD44 is a multifunctional protein involved in cell adhesion, migration and drug resistance, with a critical role in cell signaling and cell-ECM interactions in cancer. EMMPRIN (CD147) can modify the TME by activating proteases, inducing angiogenic factors in tumor and stromal cells, regulating tumor cell growth and survival and MDR. Sun et al. found that t MGr1-Ag/37LRP ligation-induced adhesion participated in protecting gastric cancer cells from a number of apoptotic stimuli caused by chemotherapeutic drugs [26]. MGr1-Ag can prompt CAM-DR through interaction with laminin while MGr1-Ag/37LRP, as a receptor for ECM components, may interact with phosphorylated FAK to activate downstream signaling pathways PI3K/AKT and MAPK/ERK. They also found that inhibiting the expression of MGr1-Ag/37LRP by monoclonal antibodies, siRNA and antisense oligonucleotides can significantly increase the sensitivity of chemotherapeutic drugs and reverse MDR.

## 3. Cascade Response Nano-Delivery System

It is the ultimate goal of the drug delivery system (DDS) to deliver anticancer drugs and release them into the tumor effectively. This goal remains challenging due to the complexity of biological barriers. The typical drug delivery of nanocarriers from the intravenous injection site to the cytosol of a tumor cell requires a five-step cascade, including: (1) circulation, (2) accumulation, (3) penetration, (4) internalization, (5) drug release, or a CAPIR cascade [27]. The overall delivery efficiency (Q) of the system is a product of the five-step efficiencies (QC, QA, QP, QI and QR). Therefore, to obtain high delivery efficiency, it is necessary to balance the efficiencies of all the steps and ensure that none of them is too low. The tumor’s enhanced permeability and retention (EPR) effect or physical targeting effect greatly promotes the accumulation of nanocarriers at the tumor site [28]. In addition, some unique characteristics of the TME, such as slight acidity (pH 6.5–7.2), overexpression of proteins and enzymes, and hypoxia, make nanocarriers responsive to internal and external stimuli more effective [29]. Responsive nanocarriers can overcome a variety of biological barriers in cascade delivery, as well as multi-targeted and multi-stage responsive release of cancer therapeutic drugs, making drug release selective and controllable. Here we mainly discuss the superiority of cascade delivery from two aspects: cascade targeting and cascade release.

### 3.1. Accurate Cascade Targeting

**Figure 1:**
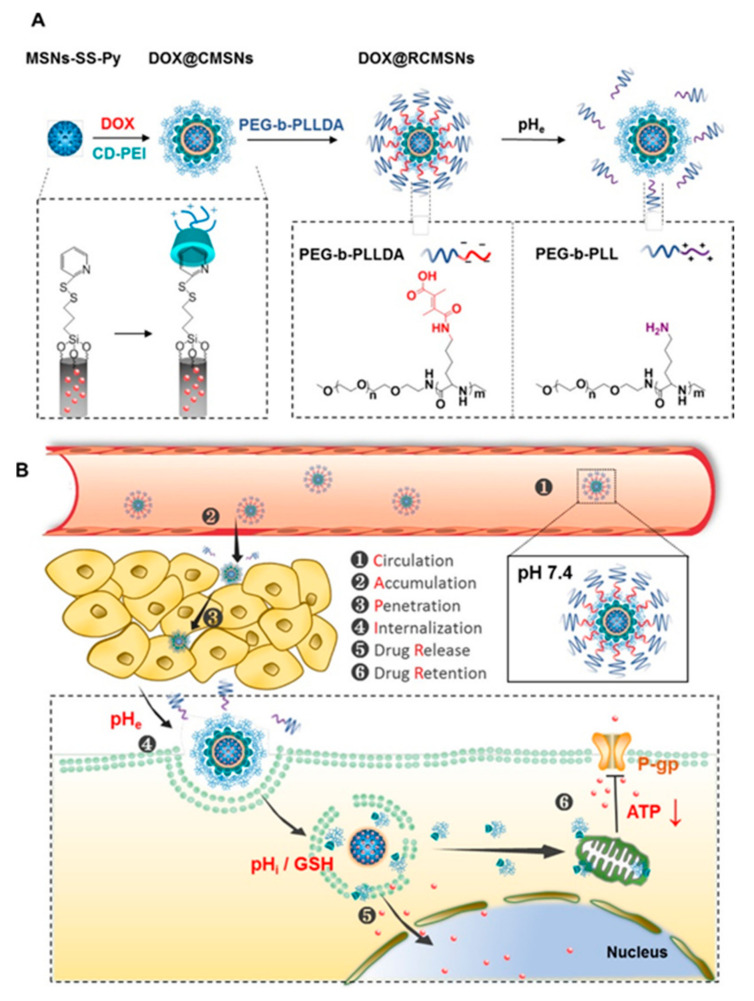
(**A**) Schematic showing the synthetic procedure of the sequentially responsive nanosystem (DOX@RCMSNs). (**B**) Schematic illustration of DOX@RCMSNs overcoming the cascaded bio-barriers and the mechanism of action in tumor cells. Adapted from [30].

Nanocarriers with cascade targeting ability can accurately locate tumor cells or specific organelles, greatly reducing the effect on normal cells of the body and improving the efficacy of tumor treatment. These modified nanocarriers can actively or passively target tumor cells through biological barriers. Some nanocarriers with cascade targeting capabilities can respond to stimuli in TME or external stimuli. This allows nanocarriers to be better designed to suit various changes in tumors. For example, Liu et al. designed a stealthy, sequentially-responsive doxorubicin (DOX) delivery nanosystem (RCMSNs), which was composed of extracellular-tumor-acidity responsive polymer shell (PEG-b-PLLDA), pH/redox-dual responsive mesoporous silica nanoparticles-based carriers (MSNs-SS-Py), and cationic β-cyclodextrin-PEI (CD-PEI) gatekeepers (Figure 1) [30]. Therefore, PEG-b-PLLDA corona makes RCMSN invisible and prolongs blood circulation time. When it reaches the tumor, the extracellular acidity will degrade PEG-b-PLLDA and reverse the surface charge of the nanosystem to positive, thereby greatly improving the tumor accumulation, penetration and internalization of RCMSNs. In cancer cells, CD-PEI gatekeepers unload DOX in response to intracellular acidity and glutathione, and functionally act as a P-gp inhibitor, inhibiting the efflux activity of P-gp by weakening the production of ATP to synergistically reverse MDR. This sequence-responsive nanosystem with stealth ability, charge conversion ability and convenient P-gp inhibitory activity is an effective biological barrier breakthrough drug carrier, which can effectively carry DOX into the cell without causing systemic toxicity. In addition, some cascade-modified nanocarriers can break under the acidic conditions of the TME or the tumor endosome. For example, Liu’s group prepared a TME cascade pH-responsive DDS (HMSNs-bCD/Ada-PEG@DOX) for tumor therapy [31]. Under the weak acid conditions of the TME (pH 6.8), the benzoicimine bonds between PEG and Ada was cleaved to promote cell uptake. Subsequently, the boronic acid-catechol ester bonds linkers were further hydrolyzed under the condition of a lower endosomal pH (4.5–6.5) for intracellular drug delivery, resulting in effective cell apoptosis. Therefore, rational use of TME and intracellular characteristics can construct an efficient DDS.

**Figure 2:**
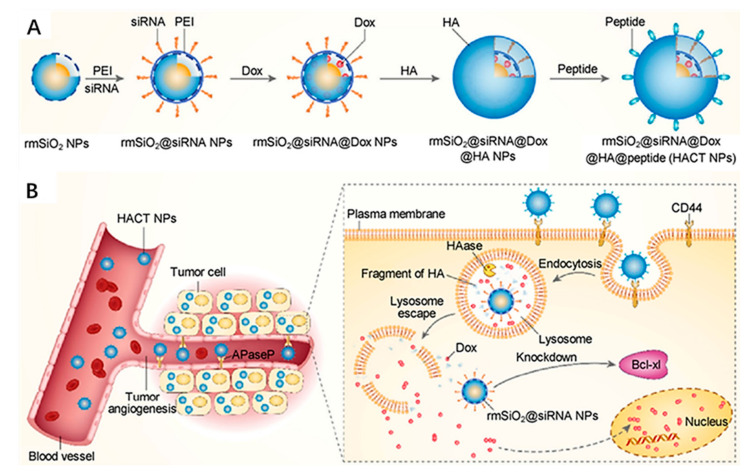
(**A**) Schematic diagram of the construction of HACT NPs with the cascade of two targeting agents (HA and peptide) and two cancer therapeutic agents (siRNA and Dox). (**B**) Schematic illustration of HACT NPs for the treatment of CTGF-overexpressing breast cancer. Adapted from [36].

The strategy of combining of drug delivery with external stimulation also has considerable advantages in the enhancement of drug positioning for effective cancer therapy. For example, Zhao et al. designed and constructed a transferrin (Tf)-conjugated photothermal nanoplatform composed of gold nanoshell-coated rod-like mesoporous silica NP (Tf-GNRS) for the delivery of the chemotherapeutic drug gemcitabine (GEM) to treat pancreas cancer [32]. This nanoplatform provides cascade tumor targeting methods, namely photothermal targeting and molecular targeting, and the combined photothermal and chemotherapy. After inducing the tumor site-specific photothermal effect, the local blood perfusion and vascular permeability of the tumor are enhanced, which improves the accumulation and penetration of Tf-GNRS in the TME. In the in vivo pancreatic tumor model, the cascade targeting effect of the plasma nanoplatform is confirmed, and it has positive feedback amplification of the antitumor efficacy. Compared with the photothermal effect or the Tf-targeting effect, this cascade targeting effect has stronger tumor cell positioning. In addition, cascade nanocarriers with multi-targeting molecular modification have positive positioning capabilities. In the research of multiple targeting nanocarriers, the CD44 receptor is a frequently studied target [33,34,35]. Ding et al. used rattle mesoporous silica (rmSiO_2_) coating and modifying HA and PEGA-PVEC peptides as siRNA and DOX delivery carriers for the treatment of breast cancer [36]. The nanoplatform is capable of targeting vascular markers and CD44 overexpressed on the surface of cancer cells through peptides and HA, allowing access to breast cancer cells through multiple barriers and achieving high selectivity for breast cancer cells (Figure 2). These cascaded targeting nanocarriers provide a nanomedical platform for highly precise transportation of multiple therapeutic agents in a synergistic treatment and strategy to overcome the drug resistance in specific cancers.

**Figure 3:**
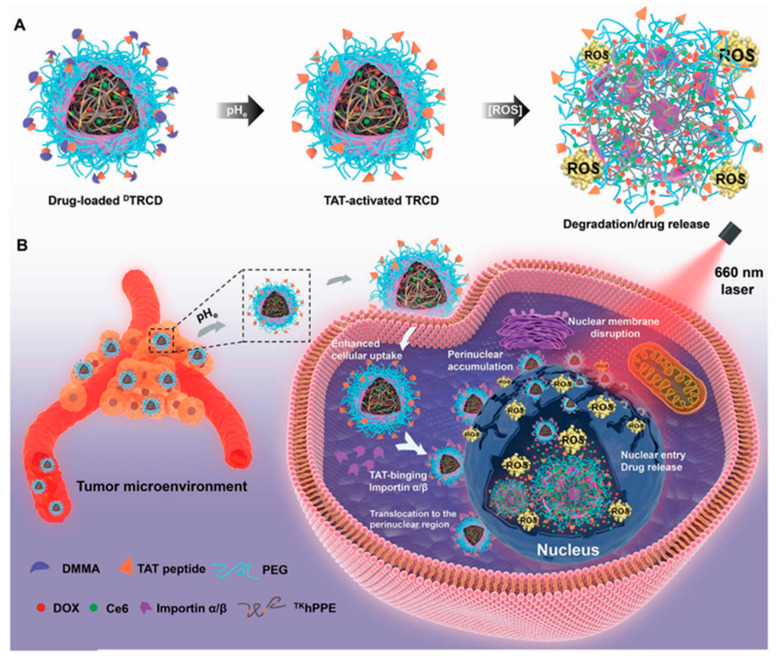
(**A**) Schematic diagram of the pHe/photosensitive decomposition of DTRCD. (**B**) Schematic illustration of the cascade nucleus-targeted drug delivery strategy of DTRCD. Adapted from [45].

Accurate delivery of therapeutic molecules to target organelles in cells can improve the therapeutic effect and achieve precise treatment. Compared with traditional treatments, this precise targeted treatment can reduce drug dosage and side effects, and avoid multidrug resistance [37,38,39]. Therefore, many works have been committed to designing active tumor targeting and specific organelle positioning functions, which can achieve cascade delivery of cargos into intracellular specific organelles [40,41]. Therefore, in order to improve the anticancer effect, the cascade targeted organelles mainly target the nucleus and mitochondria [37,41,42,43,44]. For example, Cao et al. used thioketal crosslinked polyphosphoester (PPE) NPs decorated with a pHe-sensitive transactivator of transcription (TAT) to rationally prepare a pHe/photo dual-sensitive nanocarrier (DA-masked TAT-decorating reactive oxygen species (ROS)-sensitive Ce6/DOX-loaded hyperbranched NPs (DTRCD)), (Figure 3) [45]. DTRCD prolongs circulation by masking the targeting effect of its TAT peptide, and then reactivates the TAT peptide due to its response to pHe, which further enhances tumor cell uptake and promotes transport to the perinuclear region. Next, under 660 nm laser irradiation, DTRCD can generate ROS through the encapsulated chlorin e6 (Ce6), which not only disrupts the nuclear membrane to allow entry into the nuclei, but also triggers the release of DOX in the nucleus. This cascade nuclear targeted DDS can greatly improve the efficiency of drug treatment, and reduce side effects and multidrug resistance. In addition, targeting mitochondria and nucleus double key sites has been increasingly used in order to achieve a greater anti-proliferation effect [46]. This specific DDS can not only effectively deliver drugs to the site of action, but also activate the intracellular apoptotic cascade through multiple mechanisms, thereby potentiating the antineoplastic effect. It was reported that mitochondria-to-nucleus cascade dual organelle targeted PSs have been developed to combat cancer with good effect [47]. This strategy allows the same number of PSs to be used twice, and the genes in the mitochondria and the nucleus are damaged by photosensitization. This will allow us to use limited amounts of PSs for a maximized killing effect, which is also beneficial for the overall biocompatibility of the PSs when a low amount is needed.

### 3.2. Cascading Response Release

Poor cell uptake of drugs, the inability to release adequate drugs at tumor sites, and inherent MDR are the main obstacles limiting the efficacy of tumor therapy. Due to the microenvironment limiting the delivery and penetration of particles into the tumor, the nanocarriers with TME-responsive capacities guarantee tumor-selective treatments with improved biosafety. In particular, a cascaded multi-stage DDS can ensure accurate and selective delivery of goods to targeted sites and effective release. A variety of cascaded release nanoplatforms have been designed to deliver drugs to specific sites using internal or external stimuli such as decreased pH in TME [31] or laser irradiation [48] to obtain anticancer effects.

#### 3.2.1. Internal Stimulus-Triggered Cascade Release

**Figure 4:**
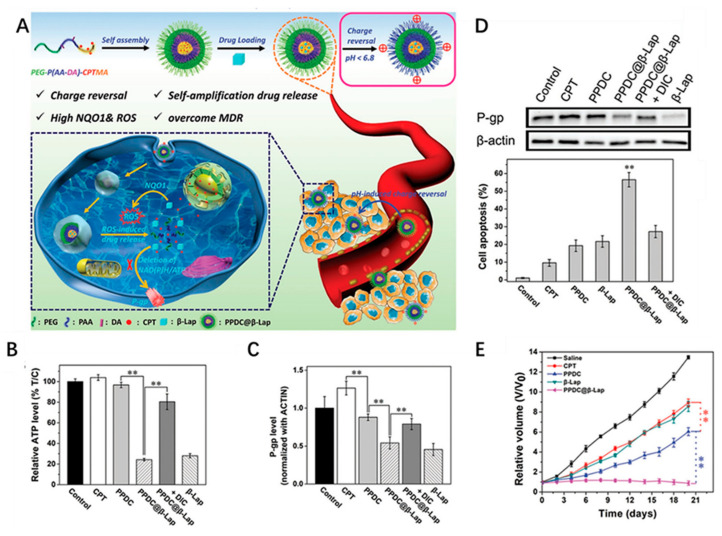
(**A**) Schematic illustration of cascade self-amplifiable drug release and charge reversal PPDC system for tumor therapy. (**B**) Intracellular ATP level in MCF-7 ADR cells treated with different formulations for 4 h. (**C**) Quantitative analysis of P-gp expression after incubation of different formulations for 48 h. (**D**) Western blotting images of P-gp expression in MCF-7 ADR cells after incubation of different formulations for 48 h. β-actin was used as control. (**E**) Volume of tumor treated with different dosage forms after 21 days. (** *p* < 0.01 (*t*-test)) Adapted from [52].

The complex nature of the TME allows the nanoplatform to be a cascaded release with these stimuli, which are primarily decreased pH, ROS and enzyme sensitivity. For example, Zhang et al. designed a macrophage-membrane-coated vesicle-loaded chemotherapeutic drug paclitaxel (PTX) for tumor targeting therapy with controllable release in an acidic TME [49]. During systemic circulation, the vesicle membrane served as a concealing cloak against opsonization and reticuloendothelial system (RES) clearance and as a tumor-homing navigator to enhance tumor accumulation. In the first release stage, after the macrophage membrane completes the tumor targeting, the interstitial pH would cause the membrane-coated formulation to undergo expansion and eruption, removing the coat. Under the action of the surface-targeting peptide, the released NPs can be better absorbed by the tumor. In the second release stage, the encapsulated PTX would be released from the NPs in response to the intracellular pH of the tumor cells. This cascade step-by-step release strategy can optimize the drug release kinetics in the TME and improve drug delivery efficiency and biocompatibility. Furthermore, tumor cells have higher levels of ROS than normal cells, which is commonly used as a condition for cascade release. Various ROS-responsive copolymers comprising of oxidation-labile groups such as thioketal, alkylene sulfide, and boronic ester have been extensively investigated to construct DDS for tumor therapy [50,51]. For example, Dai et al. constructed a self-amplifiable drug release system with charge reversal ability by loading β-lapachone in a pH/ROS cascade-responsive polymeric prodrug micelle polyethylene glycol (PEG)-P(2-aminoethyl methacrylate hydrochloride (AA)-DA)-camptothecin conjugated hydroxyethyl methacrylate-oxalyl chloride (CPTMA) (denoted as PPDC@β-Lap) [52]. In the weak acidic TME, the surface charge of the micellar system will reverse, which can increase the uptake of tumor cells. Subsequently, the cascade-responsive micellar system can dissociate and induce the release of β-lapachone and camptothecin (CPT) in ROS-rich intracellular environments (Figure 4A). In addition, released β-lapachone can produce ROS under the catalysis of nicotinamide adenine dinucleotide (NAD)(P)H:quinone oxidoreductase-1 (NQO1) and consume ATP, induce self-amplifiable disassembly of the micelles and drug release and suppress drug efflux, and finally overcome MDR (Figure 4B–D). Interestingly, this study can not only exert the synergistic effect of oxidation-chemotherapy, but also shows the characteristics of pH-responsive charge-reversal, upregulation of tumoral ROS level, self-amplifying ROS-responsive drug release and so on, which can achieve potent antitumor efficacy.

**Figure 5:**
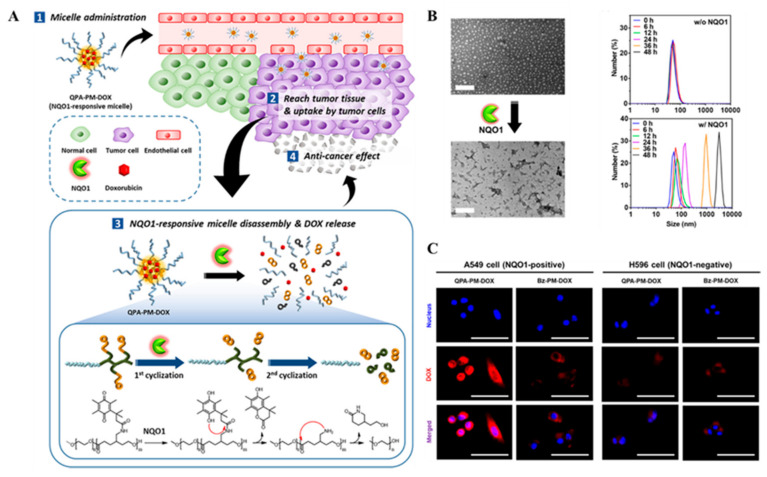
(**A**) Schematic representation of NQO1-responsive drug delivery system and drug release. (**B**) TEM images of the micelles after enzyme-mediated disassembly (scale bar: 200 nm) and time-dependent DLS size distribution of QPA-P micelles upon incubation with NQO1 enzymes. (**C**) Intracellular DOX release from QPA-PM-DOX and Bz-PMDOX in A549 (NQO1 positive) and H596 (NQO1 negative) observed by CLSM (scale bar: 50 μm). Adapted from [62].

Certain enzyme overexpressions in tumor cells were detected. It has become a new method to use enzymes as stimuli for intelligent cascade drug release in site of action [53]. Enzymes can trigger the release of drugs and biosensors through special enzyme reaction materials. Enzyme-triggered cascade drug release mechanism is mainly involved in hydrolases [54,55,56] and oxidoreductase enzyme [57]. Among them, NQO1 is overexpressed in some tumors, which maintains redox homeostasis and inhibits oxidative stress by detoxifying highly active quinones [58]. In particular, the activity of NQO1 in lung cancer and liver cancer cells increased 50 times compared with normal cells. NQO1 overexpressed in tumor tissue has been widely used in the development of stimuli-responsive DDS [59,60,61]. For example, Park et al. synthesized an amphiphilic block copolymer (QPA-P), which was triggered by NQO1 to depolymerize QPA-locked polycaprolactone (PCL) and poly (ethylene glycol) (PEG) as hydrophobic and hydrophilic components, respectively [62]. The QPA-P formed self-assembled micelles in aqueous conditions to deliver DOX. The results showed that NQO1 catalyzed the depolymerization of QPA-locked PCL through a cascade two-step cyclization process, that is, NQO1 enzyme reduced the quinone group of QPA-P to hydroquinone and then the generated hydroxyl group undergoes nucleophilic attack at the carbonyl group of the amide bond, triggering the first cyclization process and releasing the pendant group as a lactone moiety. Secondly, the exposed amino groups initiated the second cyclization process by nucleophilic attack on the ester bond in the main chain of PCL, and eliminated the lactam part of PCL (Figure 5A). This cascade two-step cyclization depolymerization process can remove the hydrophobic blocking segment, eventually induce the micelle structure dissociation, and trigger the release of DOX on target cancer cells (Figure 5B,C). In addition, in situ glucose oxidase (GOx)-catalyzed glucose oxidation can increase intracellular H_2_O_2_ concentration, which is also used to design tumor specific carrier cascade release. For example, Cheng’s group built a biomimetic cascade nanoreactor (named Mem@GOx@ZIF-8@BDOX) [63]. The nanoreactor assembled tumor cell membrane and GOx onto zeolitic imidazolate framework (ZIF-8) and loaded H_2_O_2_-sensitive BDOX. Therefore, biomimetic membrane camouflage affords superior immune evasion and homotypic binding capacities, which significantly enhances the preferential accumulation and absorption of tumor targeted drug delivery. Moreover, cascade catalysis can enhance glycolysis of GOx starvation therapy by controlling the disintegration of acid responsive ZIF-8 and the release and activation of H_2_O_2_ responsive BDOX. GOx can immobilize intracellular glycolysis, cut off glucose supply and metabolic pathways for starvation therapy, increase H_2_O_2_ concentration, and achieve TME regulation. This kind of bionic nanoreactor has the advantages of efficient drug delivery and long-term accumulation, significantly improving the therapeutic effect and reducing adverse drug reactions.

#### 3.2.2. External Stimulus-Triggered Cascade Release

**Figure 6:**
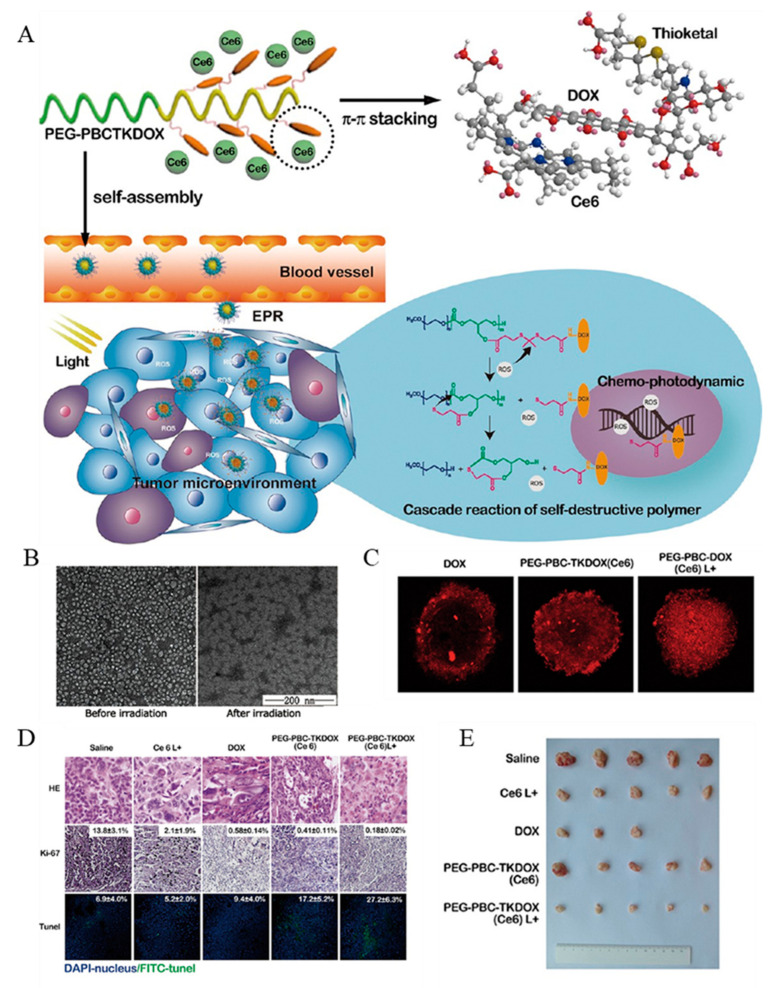
(**A**) Schematic of the cascade reaction of self-destructive polymeric nanomicelles. (**B**) zeta-potential of Ce6-loaded PEG-PBCTKDOX nanomicelles. (**C**) Penetration of DOX in MCF7/ADR 3D cell spheroids. (**D**) Tumor images of H&E staining, Ki67 immunohistochemistry, and TUNEL assay after the treatment depicting morphology, proliferation, and apoptosis, respectively. (**E**) Final tumor volume images after different drug treatment. Adapted from [4].

Light is the main stimulus to study the cascade drug release controlled by external stimuli. At present, many studies have applied light-activated nanomaterials to DDS, which would respond to drug release under light stimulation [64,65,66]. Cascaded nano delivery systems initiated by light stimulation often combine with other stimuli to improve tumor selectivity. For example, He et al. reported a cancer-targeting vehicle in response to cascaded external (light) and internal (hypoxia) dual triggers [48]. The nanoplatform was self-assembled by polyethyleneimine nitroimidazole (PEI-NI) into micelles, which were further loaded with hyaluronic acid-Ce6 (HC) and DOX. The NPs can accumulate in the tumor site through EPR effect, and can be endocytosed by tumor cells through HA binding to over-expressed CD44 on cancer cells surfaces. Under 660 nm light irradiation, high levels of ROS were generated in the tumor, which greatly enhanced the hypoxic levels, induced NPs dissociation and, accordingly, DOX release. This dual trigger DDS improves the selectivity of drugs, reduces the side effects, and renders promising applications for the programmed combination of chemotherapy and PDT. Similarly, the self-destructive polymeric nanomicelles were synthesized by connecting polycarbonate with thioketone bond which was sensitive to ROS to load PS Ce6, and DOX was loaded by π-π superposition (Figure 6A) [4]. Light given in PDT treatment not only excites Ce6 to produce cytotoxic ROS, but also spatiotemporally activates a cascade reaction to release the loaded DOX (Figure 6B,C). This self-degrading system can facilitate cellular uptake, endosomal escape, and nuclear distribution of the DOX, as well as reduce the side effects and improve the anticancer effect (Figure 6D,E).

## 4. Activated Cascade Reactions to Enhance ROS-Induced Cancer Therapy

ROS comprise of a family of short-lived molecules such as singlet oxygen (^1^O_2_), hydroxyl radical (•OH), and superoxide anion (O_2_^•−^) [67]. ROS production has been implicated in mediating chemotherapy or radiotherapy responses via its effects on downstream cell survival or death signaling cascades. The mechanism of ROS on tumor cells is complex. It not only plays a role in destroying redox homeostasis, but also destroys macromolecules that maintain cell life. There has been plenty of research on ROS-induced cancer therapies [16,68]. However, in the treatment of ROS-induced cancer, due to the lack of endogenous resources or specific microenvironment, the internal tumor is facing the dilemma of treatment deterioration. Therefore, by centralizing TME and triggering specific cascade reactions with external stimuli, we can make full use of internal resources to produce ROS, so as to achieve good therapeutic effect. Compared with traditional therapy, this method can make full use of the existing conditions to produce ROS in specific parts of the tumor and improve the therapeutic effect. At present, a large number of cascade reactions using different mechanisms have been used in cancer treatment. Most of these mechanisms revolve around two aspects; inducing the increase of ROS content and reducing the consumption of ROS.

### 4.1. Enzyme-Based Cascade Enhances ROS-Induced Cancer Therapy

**Figure 7:**
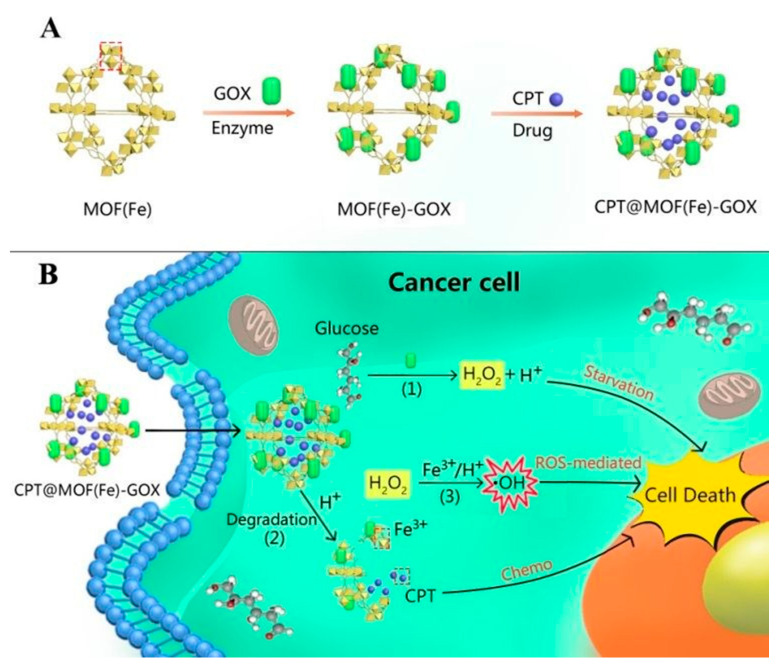
(**A**) Synthetic procedure of CPT@MOF(Fe)-GOX. (**B**) Schematic illustration of CPT@MOF(Fe)-GOX via a cascade reaction (1–3) in cancer cells. Adapted from [76].

Chemodynamic therapy (CDT) is a therapeutic method that can induce ROS in tumor cells and break the balance of intracellular redox/oxidation state [69,70,71]. However, the progress of CDT excessively depends on the reaction conditions, such as the content of H_2_O_2_, which is limited in TME [72,73]. This fact has been recognized as one of crucial obstacles for ensuring the anticancer efficacy of current CDT. Nanozyme-based CDT has become an effective anticancer method because of its small side effects and lack of requirement for exogenous energy [74]. Constructing a therapeutic system with enzyme delivery ability and triggering cascade reaction in tumor cells can significantly improve the efficacy of CDT. For example, Fang et al. synthesized a nanoscale Co–ferrocene metal–organic framework (Co-Fc NMOF) with high Fenton activity, and combined it with glucose oxidase (GOx) to construct a cascade enzymatic/Fenton catalytic platform (Co-Fc@GOx) for enhanced tumor treatment [75]. In this system, Co-Fc NMOF not only acts as a versatile and effective delivery cargo of GOx molecules to modulate the reaction conditions, but also possesses excellent Fenton effect for the generation of highly toxic •OH. In addition, Co-Fc NMOF can transfer GOx to catalyze endogenous glucose to produce gluconic acid and H_2_O_2_, which, in turn, favors the Fenton reaction of Co-Fc NMOF, and enhances the generation of ROS. This study provides an effective method for cancer prevention, which can effectively regulate the microenvironment of tumors and synergetic treatment of cancer. Liu et al. Modified GOx to the surface of Fe-based metal organic framework (MOF(Fe)) and reacted with CPT cascade loaded into MOF(Fe) cavity to form a synergistic cancer starvation/ROS-mediated/chemotherapy (Figure 7) [76]. This cascade three-mode combination therapy utilizes TME, such as over expressed glucose transporters and acidic environments, to kill cancer cells without external intervention, and has potential tumor specificity.

### 4.2. Glutathione Cascade for Enhancing Cancer Therapy

**Figure 8:**
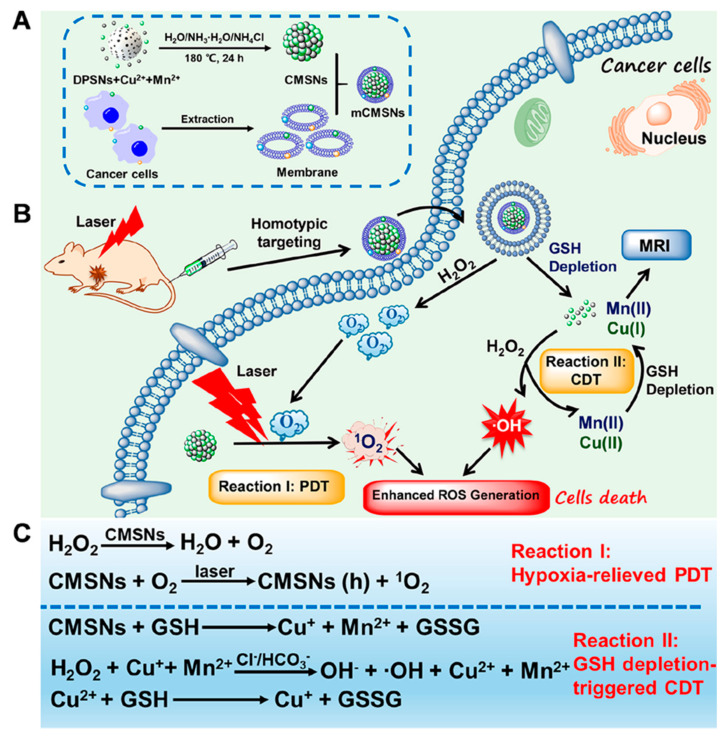
(**A**) Synthetic procedure of mCMSNs. (**B**) Schematic illustration of therapeutic mechanism of mCMSNs for PDT under Laser. (**C**) Chemical mechanism of GSH-triggered CDT and MRI. Adapted from [86].

GSH is an important antioxidant in cells, which protects cells from various oxidative damages. When GSH is overexpressed in tumor, it will limit the rise of ROS level to maintain redox homeostasis, resulting in resistance to ROS-induced cancer therapy [77]. Therefore, the reduction of GSH in TME is of great significance for promoting ROS production and ROS-induced tumor treatment. In recent years, a large number of studies have reported a variety of cascade reactions involving GSH-eliminating and catalytic ROS-generating cascade process for enhancing ROS-induced cancer treatments [78,79,80,81,82]. GSH is involved in the cascade of ROS-induced tumor therapy through various exhaustion ways. For example, GSH can be reduced into glutathione disulfide (GSSG) by metal oxides. Zhen et al. developed a new iridium oxide (IrOx) nanozyme with intrinsic multienzyme mimetic activities similar to natural catalase, peroxidase, and oxidase [83]. IrOx could continuously consume GSH through self-cyclic valence alternation of Ir^IV^ and Ir^III^ to break the antioxidation defense system of the tumor. In addition, GSH can be absorbed by the binding of active metal sites with GSH. For example, Zhang et al. reported a nano-metal–organic framework Cu^II^-metalated porphyrinic MOF (MOF-2) based on Cu^II^ as the active center for PDT [84]. Under light irradiation, MOF-2 can produce a high level of ROS, and absorb GSH in cells, which can further increase the concentration of ROS and accelerate the apoptosis of cells, thus enhancing the effect of PDT. Yang et al. synthesized seven types of bimetallic nanoparticles using metal organic framework (MOF) as stable host [85]. Among them, Cu-Pd@MIL-101, with an alloy loading of 9.5 wt% modified by PEG (9.5% CPMP), is found to exhibit high GSH depletion. This work has provided a credible strategy for constructing nanozymes with an excellent activity, and may pave a new way for the design of enhanced tumor CDT treatment. The integration of ROS-involved PDT and CDT holds great promise for enhanced anticancer effects. Liu et al. reported that biodegradable cancer cell membrane-coated mesoporous copper/manganese silicate nanospheres (mCMSNs) have the abilities of homotype targeting cancer cell lines, and they enhance the production of ROS through ^1^O_2_ production and GSH-activated Fenton reaction (Figure 8) [86]. Under 635 nm laser irradiation, mCMSNs can alleviate the tumor hypoxia microenvironment by catalyzing the decomposition of H_2_O_2_ into O_2_ and further reacting with O_2_ to produce toxic ^1^O_2_. GSH-mediated biodegradation of mCMSNs can simultaneously produce Fenton-like Cu^+^ and Mn^2+^ ions, and deplete GSH to produce effective •OH. In tumor, the destruction of hypoxic environment and the consumption of GSH can destroy TME and cell antioxidant defense system, which has a good anticancer effect.

### 4.3. Other Cascade Mechanisms Enhance ROS-Induced Cancer Therapy

**Figure 9:**
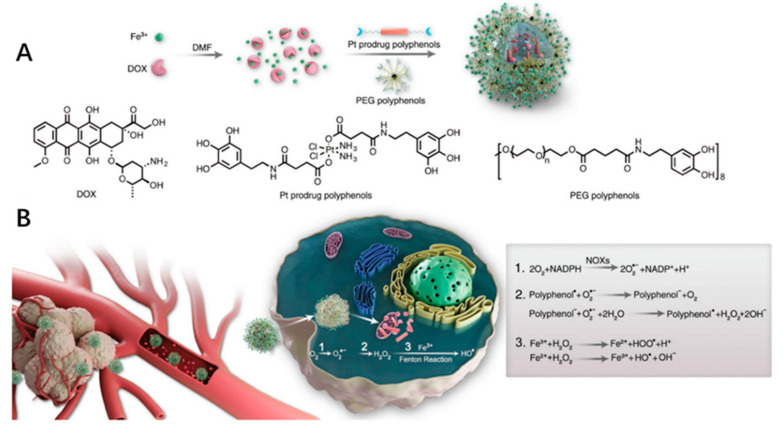
Formulation of nanoparticles and the ROS-enhanced chemotherapy mechanism. (**A**) Schematic of the DPPF NPs self-assembly process. (**B**) Schematic illustration of the DPPF NPs treating cancer mechanism. Adapted from [87].

**Figure 10:**
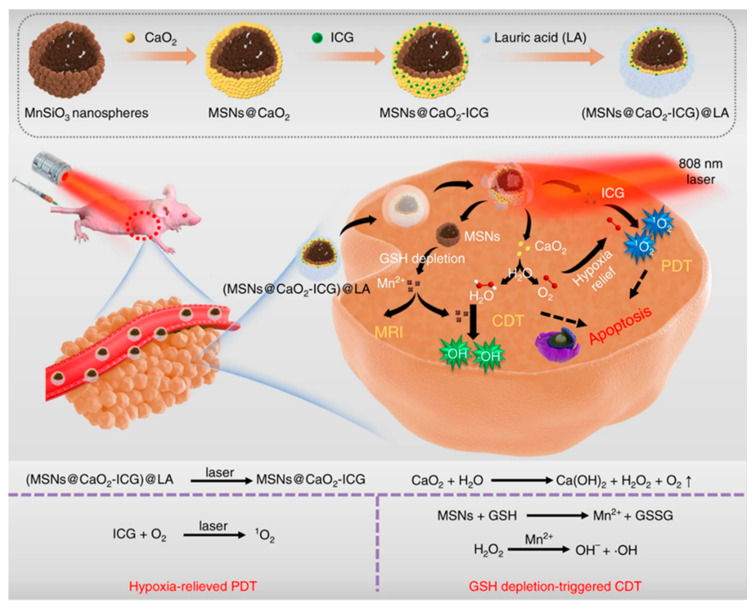
The scheme of fabrication process and therapeutic mechanism of thermo-responsive (MSNs@CaO_2_-ICG)@LA NPs for synergistic CDT/PDT with H_2_O_2_/O_2_ self-supply and GSH depletion. Adapted from [96].

It’s a common method to increase ROS content with chemotherapeutic drugs. For example, DOX and platinum drugs can activate nicotinamide adenine dinucleotide phosphate oxidases, generating superoxide radicals (O_2_^•−^) [87]. Polyphenol superoxide dismutase can catalyze O_2_^•−^ to H_2_O_2_. Finally, highly toxic HO• radicals were generated by Fenton reaction. The ROS HO• can synergize the chemotherapy by a cascade of bioreactions (Figure 9). In addition, PDT is a promising clinical cancer treatment strategy, and it is also a common means of increasing ROS content and enhancing ROS-induced anticancer strategy [88,89,90,91,92]. PDT converts O_2_ into ROS by light-activated PSs, while CDT generates cytotoxic •OH by an in situ Fenton or Fenton-like reaction between H_2_O_2_ and catalysts [93,94]. The PDT/CDT combination therapy can amplify the oxidative stress of tumor and achieve better anticancer effects than single therapy [95]. However, the effect of the TME characteristics of hypoxia and GSH depletion on ROS limits the efficiency of ROS. Therefore, expanding the production of endogenous O_2_/H_2_O_2_ or directly transporting exogenous O_2_/H_2_O_2_ into cells are two important strategies for alleviating PDT hypoxia. For example, Liu et al. reported a H_2_O_2_/O_2_ self-supplying nanoagent, (MSNs@CaO_2_-ICG)@LA, which was composed of manganese silicate (MSN)-supported calcium peroxide (CaO_2_) and indocyanine green (ICG) with further surface modification of phase-change material lauric acid (LA) [96]. Under laser irradiation, ICG produces ^1^O_2_ and releases heat to melt LA. Subsequently, the exposed CaO_2_ reacts with water to produce O_2_ and H_2_O_2_, which are used to alleviate hypoxia ICG mediated PDT and H_2_O_2_ supplied MSN-based CDT, acting as an open source strategy for ROS production (Figure 10). In addition, MSN-induced glutathione depletion protects ROS from scavenging, termed reduce expenditure. This strategy can effectively inhibit tumor growth both in vitro and in vivo, and significantly improve the multi-level production efficiency of ROS in cancer treatment involving ROS. ROS-sensitive nanocarriers responsive to TME can maintain the activity of PS in vivo, and then release or activate PS effectively under specific TME conditions. In addition, these kinds of nanocarriers often have the characteristics of self-amplification, that is, the ROS produced by itself can in turn break the ROS-sensitive chemical bonds to release more PS or drugs [97]. Wu et al. explored a nanocarrier based on oxidation sensitive polyphosphate (PPE) to co-encapsulate Ce6 and docetaxel (Dtxl) for synergistic treatment of laryngeal cancer [98]. In the process of PDT, ROS not only induces apoptosis, but also triggers the release of Dtxl through the hydrophobic to hydrophilic transition of PPE core, which leads to PDT/CDT cascade and produces synergistic anticancer effects. Additionally, the cascade PDT/CDT mediated by the oxidation sensitive nanocarrier can induce an effective anti-tumor immune response through the immunogenic cell death effect (ICD), and enhance the effectiveness of immune checkpoint blockade (ICB) antibody in inhibiting the growth of distant tumors.

## 5. Conclusions

In recent years, the cascade reaction based on nanoplatform has been increasingly applied in cancer therapy and has achieved a good therapeutic effect [99,100,101]. This review summarizes and discusses the important progress of nanotechnology in triggering specific cascade processes at tumor sites for the effective and safe treatment of cancer. The cascade reaction based on nanotechnology shows its feasibility and high efficiency in anticancer treatment. Therapeutic modalities based on cascade technology can offer advantages over conventional therapies, with selectivity and controllability in tumor targeting and drug delivery.

The recently proposed cancer treatment cascades mainly rely on endogenous characteristics (e.g., acid pH and GSH), or exogenous characteristics (e.g., light and magnetic field), so the triggered cascades depend on the availability of endogenous or exogenous characteristics. Although the cancer therapy based on the nano-drug delivery system has a good in vitro anticancer effect, the clinical research is not enough. In addition, the cascade system of TME response depends on tumor-specific triggering, but the heterogeneity of tumor interior and microenvironment, the mutation of tumor in the process, and individual differences are all factors limiting the therapeutic effect. Therefore, it means that it is necessary to design a more secure and efficient cascade system and enhance the controllability of the cascade triggering process. As a promising anticancer method, the cascade system has shown excellent performance in the field of nanomedicine, but it may still lead to tumor recurrence and metastasis. More in-depth exploration is needed to meet the needs of personalized therapy in the future.

## Data Availability

Not applicable.

## Abbreviations

PDT	Photodynamic therapy
CDT	Chemokinetic therapy
TME	Tumor microenvironment
MDR	Multidrug resistance
ROS	Reactive oxygen species
IFP	Interstitial fluid pressure
ECM	Extracellular matrix
DOX	Doxorubicin
GSH	Glutathione
PTT	Photothermal therapy
DDS	Drug delivery systems
H_2_O_2_	Hydrogen peroxide
UCNPs	Upconversion nanoparticles
ATP	Adenosine triphosphate
NIR	Near-infrared light
GC	Glycol chitosan
TK	Thioketone
PGA	Polyglycolide acid
EMT	Epithelial-mesenchymal transition
P-pg	Permeability-glycoprotein
HIF-1	Hypoxia-inducible factor-1
RES	Reticuloendothelial system
NQO1	(NAD)(P)H:quinone oxidoreductase-1
PTX	Paclitaxel
PCL	Polycaprolactone
PEG	Poly(ethylene glycol)
GOx	Glucose oxidase
PPE	Polyphosphate
Dtxl	Docetaxel
ICD	Immunogenic cell death
O_2_^•−^	Superoxide radicals
•OH	Hydroxyl radical
PS	Photosensitizer
GEM	Gemcitabine
rmSiO2	Rattle mesoporous silica
Tf	Transferrin
Ce6	Chlorin e6
CPT	Camptothecin
HC	Hyaluronic acid-Ce6
IrOx	Iridium oxide
GSSG	Glutathione disulfide
LA	Lauric acid
